# Editorial: Nutritional indicators and implications for human health

**DOI:** 10.3389/fnut.2025.1628603

**Published:** 2025-07-25

**Authors:** Virginia Maria Muniz, Juliana Dos Santos Vaz, Luciane Bresciani Salaroli

**Affiliations:** ^1^State Health Department of Espírito Santo, Vitória, Brazil; ^2^Research Group/CNPq in Epidemiology, Health and Nutrition (GEMNUT) of the Federal University of Espírito Santo, Vitória, Brazil; ^3^Graduate Program in Nutrition and Foods of the Federal University of Pelotas, Pelotas, Brazil; ^4^Graduate Program in Public Health and Graduate Program in Nutrition and Health of the Federal University of Espírito Santo, Vitória, Brazil

**Keywords:** nutritional assessment, biomarkers, malnutrition, obesity, nutritional indicators

Nutritional assessment aims to investigate nutritional parameters, risk factors and specific deficiencies, in addition to determining nutritional needs and identifying medical, psychosocial and socioeconomic determinants that may influence nutritional support therapy (1, 2). Thus, adequate hospital nutrition management, based on a careful assessment, can contribute to improving the clinical outcomes of individuals and, consequently, the quality of health care (3).

In the community setting, nutritional assessment plays a pivotal role in supporting public health policy managers, contributing to the identification and tackling of epidemic problems, such as obesity—whether resulting from inadequate diets or associated with metabolic disorders—, as well as malnutrition related to food insecurity in low-income regions (4, 5).

Heterogeneous dietary responses among individuals result from a complex interaction among genetic factors, lifestyle, socioeconomic conditions and cultural contexts. These elements highlight the need for more individualized strategies, such as precision nutrition (PN), aiming to optimize health outcomes. Hence, the use of biomarkers of food intake emerges as a promising tool to increase the accuracy of dietary surveys, providing more reliable biological information and contributing to the development of more effective nutritional interventions (6).

In this context, the Research Topic “*Nutritional indicators and implications for human health*” brings together 13 studies that present relevant evidence on the use of nutritional indicators in different approaches, both at the individual and population levels. These studies include analyses based on public and comprehensive databases, field research conducted in high- and low-income regions around the world, as well as literature reviews, which will be highlighted below.

Among the population studies, four used data from the US National Health and Nutrition Examination Survey (NHANES). Liang et al. evaluated the association between the body roundness index (BRI) and osteoarthritis (OA) in adults and identified a positive relationship between BRI and the prevalence of OA in the general population of the United States of America (USA). Zhang et al. investigating the relationship between new anthropometric indices and the risk of gallstones in US adults, observing a positive association between several anthropometric indicators and the diagnosis of this condition. Yan et al. examined the possible correlation between the intestinal microbiota and the presence of kidney stones among US adults, demonstrating the potential benefits of dietary adjustments based on the dietary index for gut microbiota (DI-GM) in reducing the incidence of kidney stones. Finally, Tan et al. analyzed the associations between nutritional risk, assessed by the Geriatric Nutritional Index (GNRI), and cognitive functions in older adults in the USA, identifying that a lower GNRI was associated with worse performance in several cognitive domains.

Other population-based studies have been conducted in communities with different levels of economic resources and provide relevant evidence for the formulation of public health policies. Liu et al., using data from the Chinese Longitudinal Healthy Longevity Survey (CLHLS), observed that improving dietary behaviors among the elderly can contribute to reducing high Body Mass Index (BMI) values to normal levels; however, the same effect was not observed in cases of low BMI. Zhao et al. applied the AHLC nutritional model (albumin + HDL-cholesterol + lymphocytes + calcium) in a Chinese community population, with good results in the early detection of sarcopenia. Two studies were conducted in communities in Ethiopia. Menber et al. evaluated the performance of the Minimum Dietary Diversity for Women (MDD-W) indicator in predicting the adequacy of micronutrient intake among lactating women in a northeastern region of the country, finding results that diverged from previous studies conducted in other contexts. Zebene et al. investigated the accuracy of mid-upper arm circumference (MUAC) in identifying different forms of thinness among adolescent girls in schools in Addis Ababa, concluding that incorporating this indicator may be especially useful in resource-limited settings.

Three studies are notably addressed in hospital research. Zou et al. analyzed the performance of six nutritional indicators in predicting the prognosis of individuals with COVID-19, identifying the most effective index for this purpose. Díaz-Amaya et al. investigated the relationship between nutritional status, assessed by means of the phase angle, and postoperative complications in pediatric individuals undergoing surgery. The results indicated that the phase angle may be a valuable marker in the prevention of surgical complications in this population. Bae et al. examined variations in the values of the Prognostic Nutrition Index (PNI) in the postoperative period of patients undergoing myocardial revascularization using the off-pump coronary artery bypass (OPCAB) method. The study highlighted the importance of monitoring the PNI in the postoperative period as a tool for screening individuals at higher risk of mortality.

Among the studies presented, two important literature reviews stand out. Lu and Li conducted a systematic review with meta-analysis on the Controlling Nutritional Status (CONUT) score and its application in individuals with hematologic malignancies, demonstrating that CONUT may have predictive value in the prognosis of some of these conditions. Singh et al. developed a review on precision nutrition, an approach that considers multiple individual factors such as age, sex, genetic and epigenetic profile, in addition to pathophysiological conditions to formulate more specific dietary recommendations. This nutritional strategy aims, among other objectives, to favor the enrichment of the desirable microbiota, contributing to the promotion of a healthy diet with potential positive impact, especially for future generations.

Overall, the findings gathered in this Research Topic highlight the relevance of nutritional indicators both in individual clinical assessment, as a tool for planning nutritional support and predicting clinical outcomes, and in qualifying hospital care. Furthermore, they highlight the strategic role of these indicators in public health, especially in addressing nutritional deficiencies in communities with limited economic resources. The studies also reinforce the association between nutritional indicators and various health conditions related to eating habits, lifestyle, and socioeconomic and cultural contexts, pointing to the importance of integrated and contextualized approaches in nutritional care at the population and individual levels.

## References

[1] Serón-Arbeloa C Labarta-Monzón L Puzo-Foncillas J Mallor-Bonet T Lafita-López A Bueno-Vidales N et al. Malnutrition screening and assessment. Nutrients. (2022) 14:2392. 10.3390/nu14122392 35745121 PMC9228435

[2] Correia MITD. Nutrition screening vs nutrition assessment: what's the difference? Nutr Clin Pract. (2018) 33:62–72. 10.1177/0884533617719669 28727954

[3] Zhang H Wang Y Jiang ZM Kondrup J Fang H Andrews M et al. Impact of nutrition support on clinical outcome and cost-effectiveness analysis in patients at nutritional risk: a prospective cohort study with propensity score matching. Nutrition. (2017) 37:53–9. 10.1016/j.nut.2016.12.004 28359363

[4] Janczy A Kaska Ł Proczko-Stepaniak M Skotnicka M Stankiewicz M Szymański M. Assessment of nutritional and low-grade inflammation status among bariatric surgery candidates in the preoperative period during the COVID-19 pandemic. Obes Res Clin Pract. (2023) 17:275–8. 10.1016/j.orcp.2023.05.007 37271701 PMC10203879

[5] Zehra M Saleem A Kazi Z Parkar S. Mid-upper arm circumference assessment and comparison with weight for length z-score in infants ≤ 6 months as an indicator of severe acute malnutrition. Cureus. (2021) 13:e18167. 10.7759/cureus.18167 34707950 PMC8530741

[6] Hinojosa-Nogueira D Subiri-Verdugo A Díaz-Perdigones CM Rodríguez-Muñoz A Vilches-Pérez A Mela V et al. Precision or personalized nutrition: a bibliometric analysis. Nutrients. (2024) 16:2922. 10.3390/nu16172922 39275239 PMC11397555

